# Expression changes in pelvic organ prolapse: a systematic review and *in silico* study

**DOI:** 10.1038/s41598-017-08185-6

**Published:** 2017-08-09

**Authors:** Maryam B. Khadzhieva, Dmitry S. Kolobkov, Svetlana V. Kamoeva, Lyubov E. Salnikova

**Affiliations:** 1 Vavilov Institute of General Genetics, Russian Academy of Sciences, 3 Gubkina str., Moscow, 119333 Russia; 2 Dmitry Rogachev National Research Center of Pediatric Hematology, Oncology and Immunology, 1 Samory Mashela str., Moscow, 117997 Russia; 3 Pirogov Russian National Research Medical University, 1 Ostrovitianov str., Moscow, 117997 Russia

## Abstract

Pelvic organ prolapse (POP) is a highly disabling condition common for a vast number of women worldwide. To contribute to existing knowledge in POP pathogenesis, we performed a systematic review of expression studies on both specific gene and whole-genome/proteome levels and an *in silico* analysis of publicly available datasets related to POP development. The most extensively investigated genes in individual studies were related to extracellular matrix (ECM) organization. Three premenopausal and two postmenopausal sets from two Gene Expression Omnibus (GEO) studies (GSE53868 and GSE12852) were analyzed; Gene Ontology (GO) terms related to tissue repair (locomotion, biological adhesion, immune processes and other) were enriched in all five datasets. Co-expression was higher in cases than in controls in three premenopausal sets. The shared between two or more datasets up-regulated genes were enriched with those related to inflammatory bowel disease (IBD) in the NHGRI GWAS Catalog. ECM-related genes were not over-represented among differently expressed genes. Up-regulation of genes related to tissue renewal probably reflects compensatory mechanisms aimed at repair of damaged tissue. Inefficiency of this process may have different origins including age-related deregulation of gene expression.

## Introduction

Pelvic organ prolapse (POP), the dropping of the pelvic organs due to the loss of normal support of the vagina, is an age-related condition associated with enormous physical and emotional discomfort for a vast number of women worldwide. In total, POP affects 40–50% of women, being one of the most common reasons for gynecological surgery^1–3^. Despite a relatively large number of scientific papers on POP, mechanisms of its occurrence remain unclear^2^, whereas understanding of POP pathophysiology is necessary for its prevention and treatment.

Expression studies provide valuable information for deciphering molecular mechanisms of diseases. Several reviews on POP have included results of the investigations of expression changes in POP mainly focusing on the genes/proteins of collagen, elastin, matrix metalloproteinases and their tissue inhibitors^4, 5^. The comparison of the expression patterns of multiple genes in different studies may be useful for understanding of disease pathogenesis at the gene level. To the best of our knowledge, no research has been performed so far to analyze all available data from expression studies in POP, on both specific gene and whole-genome/proteome levels. In order to identify new genes and biological processes implicated in POP pathogenesis, we conducted a systematic review of the expression studies and an *in silico* analysis of publicly available data sets related to POP development.

## Results

### Overview of studies assessing individual gene expression profile in POP

**Figure 1:**
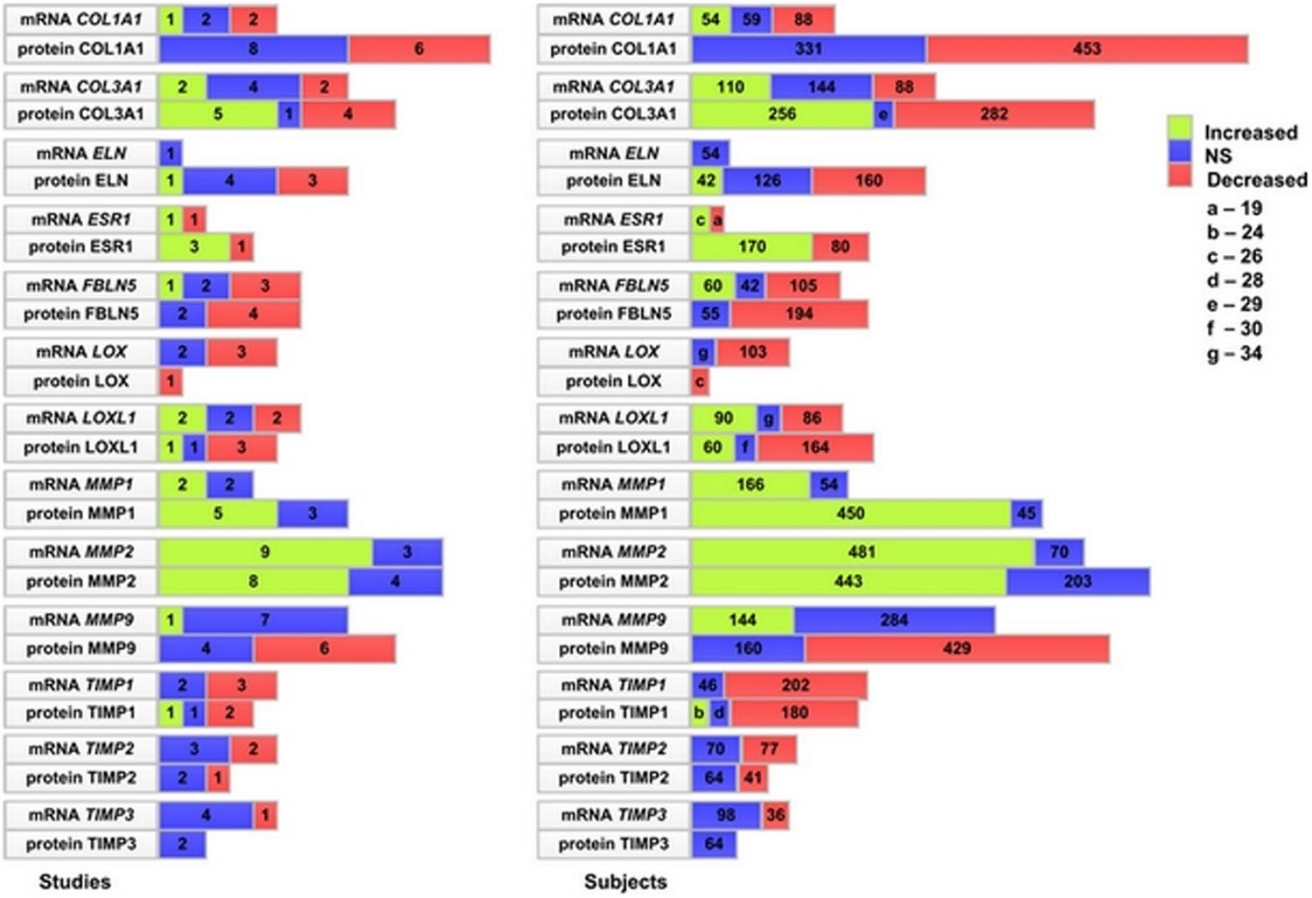
Summary of literature data on POP-related expression changes for selected genes. Numbers in boxes indicate the number of studies (Fig. 1a) and subjects (Fig. 1b) for each marker. Letter symbols (**a–g**) in the small boxes are decrypted in the right upper corner.

From a total of 465 studies found on the theme of investigation by searching PubMed, Embase and Web of Knowledge resources (Supplementary Figure S1) 78 papers were selected for data analysis (Supplementary Table S1). One hundred twenty two gene or protein products were studied, 113 of them corresponded to specific genes (Supplementary Table S1). In general, papers on associations between studied genes or proteins and POP are characterized by high heterogeneity in experimental design and data presentation. The visualization of survey data for the genes/proteins investigated in five or more studies was conducted to display proportions of data for up-regulation, down-regulation or non-significant associations for all studied genes/proteins and POP (Fig. 1). Proportions are indicated for the number of studies and for the number of subjects in these studies. The least variable results with the largest number of studies being performed were found for *MMP2/* MMP2 followed by *MMP1*/MMP1. Data for MMP1 were in agreement with results of the only meta-analysis in the field, namely with a higher expression level of MMP1 protein in POP cases in comparison with controls^6^. Decreased activity or non-significant results were mainly registered for TIMP Metallopeptidase Inhibitors, Collagen type I alpha 1, Lysyl oxidase, Fibulin 5 and Elastin. Other data appeared to be far more contradictory.

**Figure 2:**
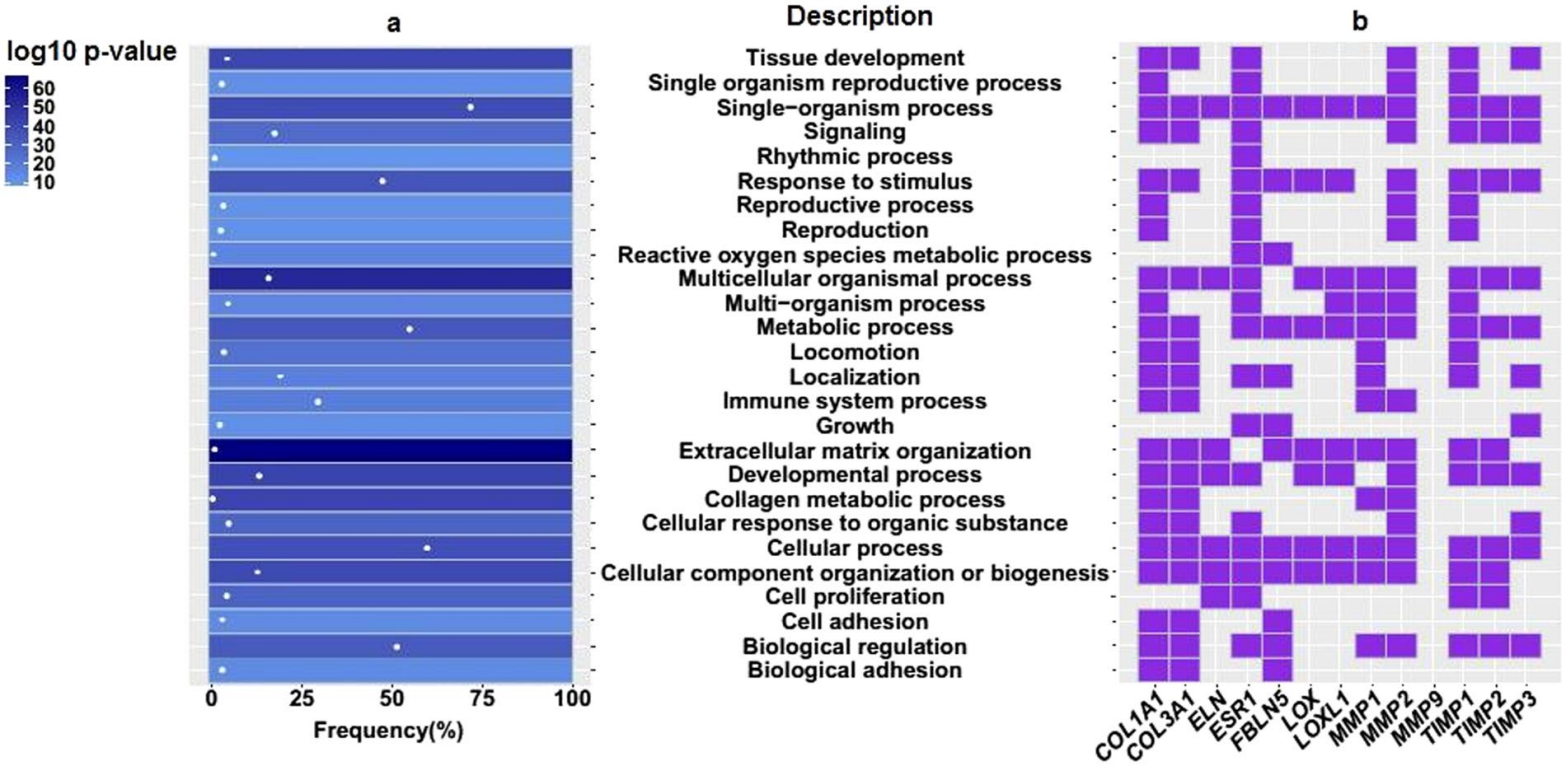
(**a**) Heat map for GO terms cluster representatives for genes considered in POP studies. (**b**) GO terms associated with the selected genes. Color indicates the user-supplied *P-* value; the term ‘frequency’ means frequency of the specific term in the underlying GO annotation database.

Next, we applied KOBAS 3.0 resource^7^ to perform GO (Gene Ontology)^8^ gene set enrichment analysis for the gene spectrum considered in the expression studies. REVIGO^9^ summary for the cluster GO representatives is provided in Fig. 2. As expected, the highest enrichment (the lowest *P*-value) was found for the term “extracellular matrix organization” which is the most specific relative to other displayed GO terms (has the lowest frequency in the underlying GO annotation database). From 13 genes presented in Fig. 1, 11 genes were related to extracellular matrix organization (ECM). These genes also contributed to the other biological processes which are indicated for the whole set of the studied genes (Fig. 2).

### Overview of whole genome/proteome studies of POP

Papers assessing the whole genome/proteome expression profile in POP did not provide consistent results (Table 1). Two studies on pubococcygeus tissue in POP patients have shown both up- and down-regulation of cytoskeletal genes or proteins^10, 11^. In full-thickness vaginal wall biopsies, genes related to smooth muscle contraction, proteolysis, response to oxidative stress, transcriptional regulation, cytoskeletal organization, and lipid catabolism were over-expressed^12^. The analysis of 34 arrays (17 round ligaments (RLs) and 17 uterosacral ligaments (USLs)) has revealed that ‘immunity and defense’ genes were up-regulated in POP^13^. Expression changes of transcriptional response and signal transduction genes associated with estrogen were detected in the USLs of POP patients^14^. Genes relevant to cell cycle, proliferation and embryonic development as well as genes related to cell adhesion were down-regulated in USLs of females with uterine prolapse^15^. In USL samples, differently expressed genes (DEG) between POP patients and controls were significantly enriched with those related to canonical Wnt receptor signaling pathway (GO term) and neuroactive ligand-receptor interaction (pathway)^16^.

**Table 1:** Characteristics of the whole genome/proteome studies of POP.

Dataset	Number of samples (age/MP status)	Analysis	DEG (DEP)	Main findings^a^	Confirmation of whole genome/proteome data	Reference
Pubococcygeus muscle in Caucasian POP patients and mixed ethnicity controls	5 cases (58.9 ± 5.5); 5 controls (45.5 ± 11.4)	Microarray gene expression	Down-regulation in POP patients: FC > 2.0, 257 genes; FC > 5, 20 genes; FC > 10, 3 genes. Up-regulation in POP patients: FC > 2.0, 479 genes; FC > 5, 18 genes; FC > 10, 2 genes.	The genes *MYBPH* (FC 24.7), *MYH3* (FC 17.4) and *COMP* (FC 6.0) were down-regulated, while smooth muscle myosin heavy chain (FC 11.8), *MLCK* (FC 5.77) and *TNC* (FC 5.1) genes were up-regulated in POP patients in comparison with controls.	No	10
Full-thickness vaginal wall biopsies from patients with POP and age-, parity -, and body mass index -matched controls^b^	5 PrM cases, 5 PrM controls	Microarray gene expression	Upregulated genes (n = 50) comprised those involved in smooth muscle contraction, proteolysis, response to oxidative stress, transcriptional regulation, cytoskeletal organization, and lipid catabolism.	The *PPP1R12A* and *ADAMTS1* genes were up-regulated 2.4 and 4-fold in cases compared to controls.	mRNA analysis for 4 matched pairs of POP/control tissues	12
USLs and RLs from mixed ethnicity patients with uterine prolapse (POP-Q stage ≥ 2) and controls	8 POP patients (5 PrM and 3 PM); 9 controls (6 PrM and 3 PM)	Microarray gene expression	In a combined analysis of expression changes in USLs and RLs, 1521 genes were up-regulated and 1193 genes were down-regulated with FC change 1.5. 249 up-regulated gene probes met FDR criteria ≤ 5.18.	After FDR correction for multiple testing, genes enriched for ‘immunity and defense’ were up-regulated in POP patients.	qRT-PCR Up-regulation in POP patients: *IL-6* (FC 9.8), *ATF3* (FC 2.6), *THBS1* (FC 3.5) and *PTGS2* (FC 2.4)	13
Pubocervical fascia from females with POP and SUI and from asymptomatic controls^b^	4 cases (PM); 3 controls (PM)	Proteomic analysis (two-dimensional electrophoresis and matrix-assisted laser desorption/ionization time-of-flight mass spectrometry)	7 proteins with an expression FC more than two (3.8 ─ 54.3-fold).	The expression levels of transgelin (FC 54.3), smooth muscle gamma-actin (FC 6.3), myosin light polypeptide 6 (FC 4.4), and alpha-1 antitrypsin precursor (FC 4.3) were higher in patients than in controls.	2-DE western blot analysis for transgelin	11
USLs from Asian women with POP-Q stage 3-4 (cases) or 0-1 (controls)	12 cases (PM), 5 controls (PM)	Microarray gene expression	143 genes were up-regulated and 87 genes were down-regulated with FC > 1.5 (*P* < 0.05).	The expression levels of *ESRRA* (FC 0.434) decreased, while the expression levels of *DAPK2* (FC 2.013), *IL15* (FC 2.1) and *STAP2* (FC 2.392) increased in POP patients compared with controls.	qRT-PCR for the genes *ESRRA, DAPK2, IL15*, *STAP2*	14
USLs from patients with uterine prolapse and controls^b^	16 POP patients (15 patients PM); 9 controls (7 females PM)	Microarray gene expression	21 up-regulated and 7 down-regulated genes with FC > 2.0 were found in patients with POP compared to the control group.	Down-regulation in POP patients compared with controls after FDR correction for multiple testing: *NKX2-3* (FC 2.6) in the whole set; *KIF11* (FC 1.3) in patients with ≥ 3 deliveries compared to patients with < 3 deliveries; *UGT1A1* (FC 1.4), *SCARB1* (FC 3.2) and *NKX2-3* (FC 6.2) in PrM patients compared to PrM controls; *UGT1A1* (FC 1.1) in PM patients compared to PM controls.	No	15
USL samples of patients with stage II to stage IV POP (mean age 61 years) and normal controls (mean age 55 years)^b^	No data	RNA-seq (a HiSeqTM 2500 platform (Illumina))	After FDR-correction, a total of 81 genes had different expression patterns in POP patients and controls. Sixty-six DEGs did not differ between the POP samples with different stages	Canonical Wnt receptor signaling pathway was the most significantly enriched GO term (*P-* value = 3.33E-07), and neuroactive ligand-receptor interaction was the most significantly enriched pathway (*P-* value = 1.24E-03).	No	16

^a^Data are uncorrected for multiplicity, otherwise specified. ^b^Ethnicity is not specified.

Abbreviations: DEG, differently expressed genes; DEP, differently expressed proteins; FC, fold change; FDR, false discovery rate; PM, postmenopausal; PrM, premenopausal; RLs, round ligaments; SUI, stress urinary incontinence; USLs, uterosacral ligaments.

### Overview of available GEO datasets for POP

Among published whole genome studies, only the study of Brizzolara^13^ has been represented in the GEO database repository^17^. An unpublished study assessing gene expression profile in the sites of prolapsed versus non-prolapsed vaginal tissues is also available in the resource (Table 2).

**Table 2:** Characteristics of the POP datasets in the Gene Expression Omnibus (GEO) database repository.

Dataset	Reference	Tissue	Number of samples	GEO accession no; Platform	Number of DEG^a^ with FDR-corrected *P -* value < 0.05	Number of DEG^a^ with FC ≥ 2.0 and *P* - value < 0.05 (uncorrected for multiplicity)
PrM Caucasian women (cystocele POP-Q stage ≥ 2), n = 12	Citation missing	AVW (POP site) versus precervical AVW (non POP site)	12 women (24 samples)	GSE53868; GPL18142 Agilent-014850 WHGM 4 × 44K G4112F	50 (↑ 31; ↓ 19)	74 (↑ 58; ↓16)
PrM POP women, n = 5^b^	Brizzolara *et al*.^13^	RLs	5	GSE12852; GPL2986 ABI Human Genome Survey Microarray Version 2	PrM_RLs set 0	PrM_RLs set 287 (↑ 246; ↓ 41)
		USLs	5			
PrM controls, n = 6^b^		RLs	6		PrM_USLs set 0	PrM_USLs set 972 (↑ 949; ↓23)
		USL	6			
PM POP women, n = 3^b^		RL	3		PM_RLs set 0	PM_RLs set 485 (↑ 345; ↓ 140)
		USL	3			
PM controls, n = 3^b^		RL	3		PM_USLs set 2 (↑ 2; ↓ 0)	PM_USLs set 494(↑ 263; ↓ 231)
		USL	3			

^a^↑ up-regulation; ↓ down-regulation; ^b^Mixed ethnicity.

Abbreviations: AVW, anterior vaginal wall; DEG, differently expressed genes; FC, fold change; FDR, false discovery rate; WHGM, whole human genome microarray; PM, postmenopausal; PrM, premenopausal; RLs, round ligaments; USLs, uterosacral ligaments.

Since the focus of our study was to find consistent patterns of gene expression profiles in different POP-related datasets, we subjected the whole dataset of Brizzolara^13^ stratified by the sort of ligaments (USLs or RLs) and the menopausal status (premenopausal (PrM) or postmenopausal (PM)) to the analysis. We were guided by the fact that the USLs, being the main supportive structures of the uterus and vagina^18^, have demonstrated higher tensile biomechanical properties (stiffness and maximum stress) than RLs^19^. These differences may be linked with distinctive features of gene expression profile. Additionally, in the study of Brizzolara^13^ USLs and RLs differed by smooth muscle cells composition; moreover, among the top 250 DEG genes only five genes coincided for USLs and RLs (the genes *F5*, *NR4A3*, *CLC*, *SLC24A4* and *GDF15* were up-regulated in both series). With regard to menopausal status, we have taken into account that expression patterns often differ between PrM and PM females^20–25^. Given that menopause is one of the main risk factors for POP, sample stratification based on menopausal status may provide better comparable results.

### GO enrichment analysis for the GEO datasets

**Figure 3:**
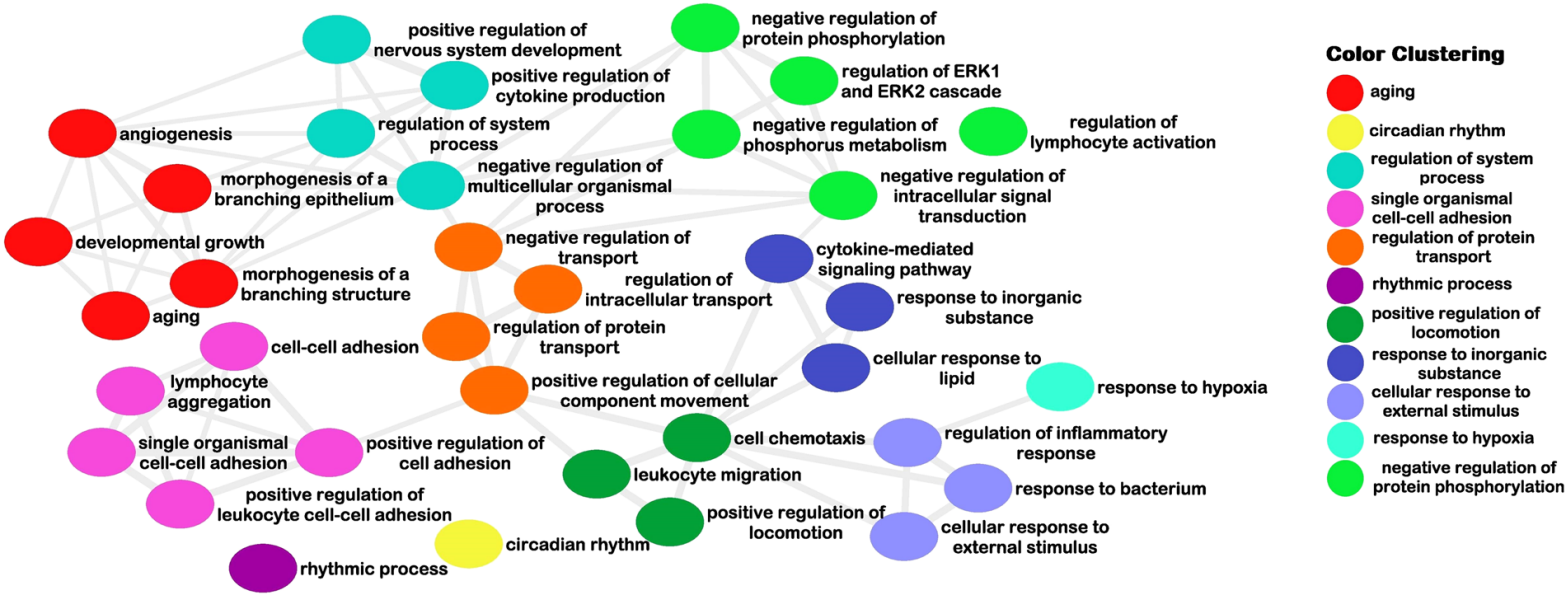
Graph for the results of enrichment analysis for five gene sets up-regulated in POP. More similar nodes are placed closer together. The line width indicates the degree of similarity between GO terms cluster representatives. Clustering by color into super clusters was obtained by using REVIGO TreeMap application.

DEG were analyzed with the KOBAS 3.0 application. Data for the series of not less than five genes associated with GO terms in the studied sets are presented in Supplementary Table S2. The number of enriched terms was noticeably larger for up- than for down-regulated genes. Biological process gene ontology terms for the five sets of over-expressed genes were summarized with the REVIGO application (Fig. 3). This analysis showed massive enrichment for genes implicated in tissue renewal and regeneration.

Up-regulated genes were associated with a number of shared terms for all five gene sets. Interestingly, among the most specific GO categories (not more than 500 background genes in the GO database) terms that include the wording ‘positive regulation’ covered biological processes related to: adhesion (n = 3), locomotion (n = 4), activation (n = 3), transport (n = 2) as well as cytokine production, MAPK cascade and nervous system development. These mechanistic considerations also provide some evidence that genes that control tissue repair are over-represented among the up-regulated genes in all data sets under study.

### A bird’s eye view on co-expression data for DEG

**Figure 4:**
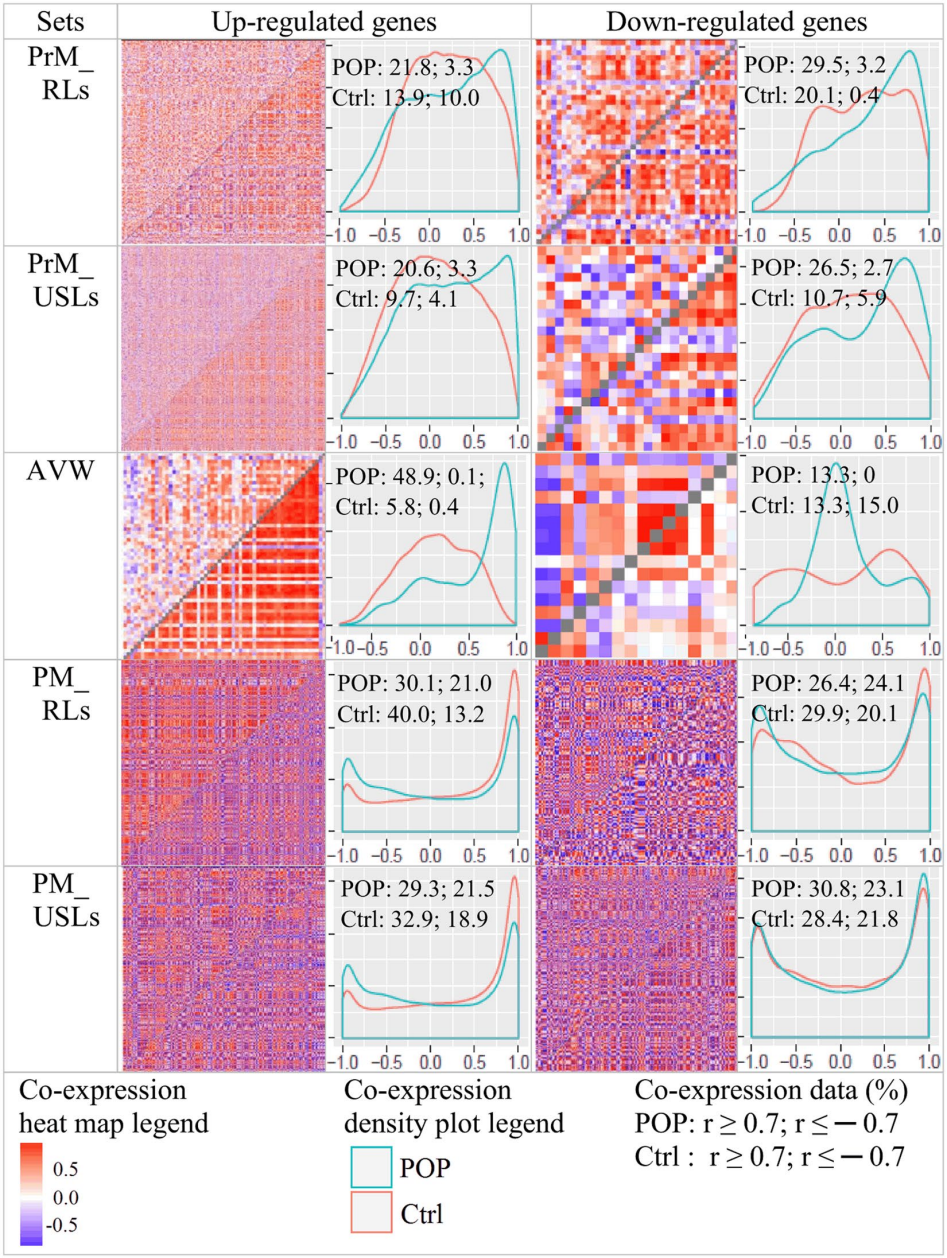
Co-expression heatmaps and density plots. Heatmaps display Pearson correlation coefficients for gene expression in controls (above the diagonal) and cases (below the diagonal). Density plots present correlation coefficients distribution for controls (blue) and cases (red) with addition of percentage of Pearson correlations (r-values) ≥ 0.7 and ≤ -0.7. Density plots x-axis: Pearson correlation coefficient (r), y-axis: density.

Co-expression analysis may give insights into altered regulatory mechanisms between disease and healthy controls, since co-regulated genes tend to exhibit similar expression patterns. Given that up-regulated genes in POP tissues were enriched with those relevant to tissue repair, we found it interesting to perform a comparative co-expression analysis. Pearson correlation coefficients were plotted in heat maps and density plots for pared genes (Fig. 4). Higher levels of co-expression in prolapsed versus healthy tissues were revealed among sets of up-regulated genes in all premenopausal groups (PrM_RLs, PrM_USLs, AVW) and down-regulated genes in the groups PrM_RLs and PrM_USLs. This observation may reflect the underlying activity of transcriptional networks, indirectly supporting the assumption on activation of regeneration processes in POP tissues; however, co-expression of down-regulated genes may reflect some intrinsic problems in realization of these processes. In postmenopausal sets (PM_RLs and PM_USLs), density plots for up- and down-regulated genes were somewhat similar, thus indicating relatively high levels of positive and negative correlations in both control and POP specimens. A possible explanation of this phenomenon is that in aging tissues disturbances in regulatory mechanisms can predominate and/or obscure the activity of the repair processes. The results may also be random due to the small samples size.

### Enrichment analysis (GO, KEGG, GWAS Catalog) for the shared gene set

A total of 142 up-regulated and 12 down-regulated genes were shared between two or more datasets (Supplementary Table S3). All top DEG in the study of Brizzolara^13^ appeared to be in the list of shared genes.

**Figure 5:**
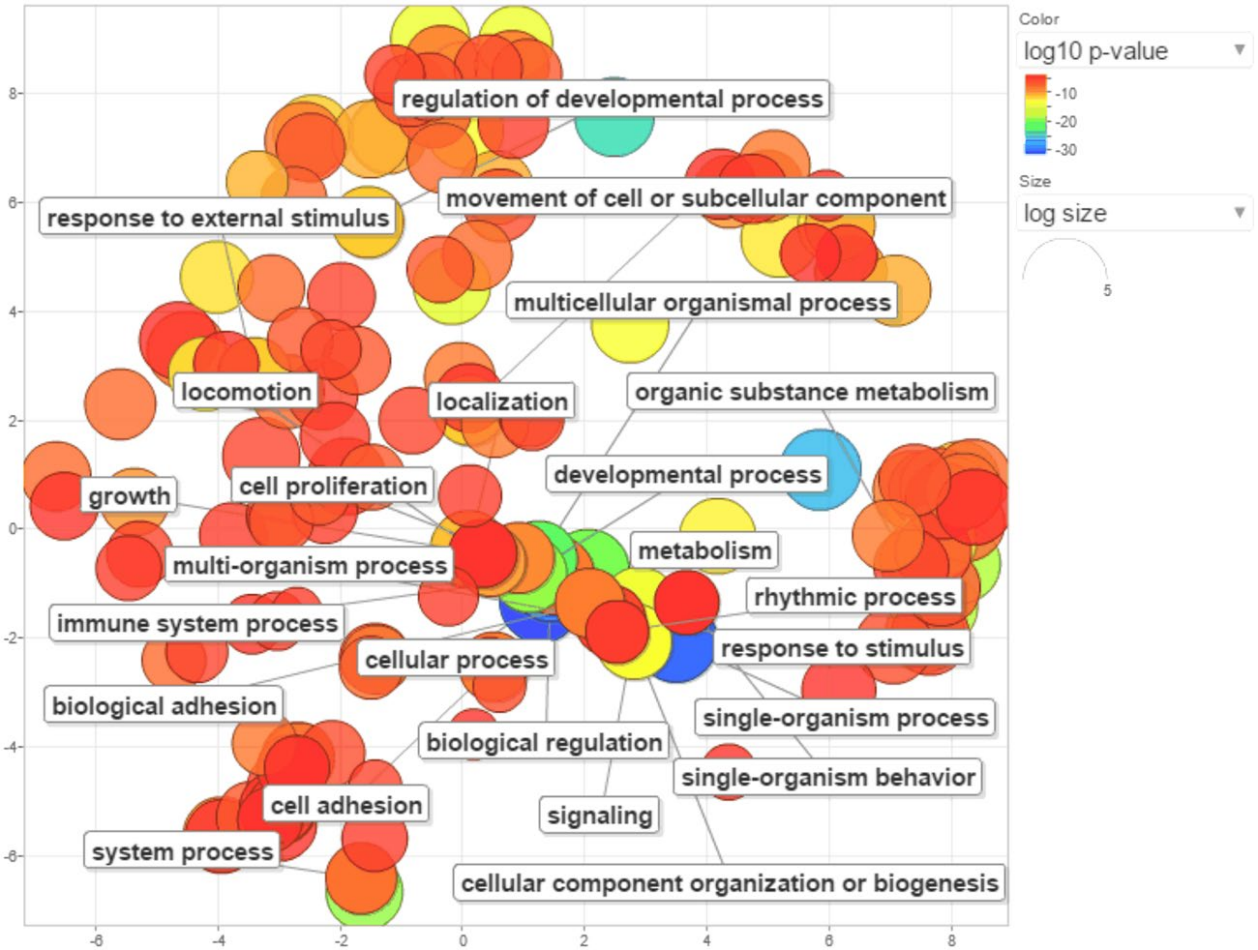
REVIGO scatterplot for GO terms cluster representatives for shared genes up-regulated in POP tissues. Bubble color indicates the user-supplied *P* - value; size shows the frequency of the GO term in the GO annotation database.

The results of GO enrichment analysis for shared up-regulated genes correlated with those obtained for the individual sets (Fig. 5). For down-regulated genes, GO analysis did not yield significant terms.

All shared up-regulated genes were tested for enrichment of metabolic pathways and association signals from GWAS data (Table 3). The enriched KEGG pathways were mainly linked with inflammatory and immune-mediated diseases. Of great interest were the results of the enrichment analysis of GWAS association data which indicated that genes associated with inflammatory bowel disease (IBD) and Crohn’s disease (one of the two main forms of IBD) were overrepresented among shared up-regulated genes. These genes (with the exception for the genes *SLC22A4, BORCS5* and *CNNM1*) are involved in ‘immune system process’ (GO term).

**Table 3:** The results of gene set enrichment analysis for shared genes up-regulated in POP tissues.

Term	Input number	Background number	*P* - value	Corrected *P* - value	Genes
**KEGG Pathway**					
Malaria (hsa05144)	7	49	1,25E-09	1,93E-07	*COMP, ICAM1, IL6, CXCL8, CCL2, THBS1, THBS4*
Influenza A (hsa05164)	8	176	3,48E-07	2,69E-05	*NLRP3, ICAM1, IL6, CXCL8, NXF3, CCL2, SOCS3, RSAD2*
Rheumatoid arthritis (hsa05323)	5	91	2,51E-05	1,19E-03	*CTSK, ICAM1, IL6, CXCL8, CCL2*
PI3K-Akt signaling pathway (hsa04151)	8	342	3,91E-05	1,19E-03	*CDKN1A, COMP, GNGT1, IL6, MCL1, PPP2R2C, THBS1, THBS4*
AGE-RAGE signaling pathway in diabetic complications (hsa04933)	5	101	4,04E-05	1,19E-03	*ICAM1, IL6, CXCL8, SERPINE1, CCL2*
Chagas disease (American trypanosomiasis)	5	104	4,62E-05	1,19E-03	*IL6, CXCL8, SERPINE1, PPP2R2C, CCL2*
Hepatitis C (hsa05142)	5	133	1,41E-04	2,43E-03	*CDKN1A, CXCL8, LDLR, PPP2R2C, SOCS3*
Hepatitis B (hsa05161)	5	146	2,15E-04	3,32E-03	*CDKN1A, EGR2, EGR3, IL6, CXCL8*
Phagosome (hsa04145)	5	155	2,80E-04	3,95E-03	*COMP, FCAR, THBS1, THBS4, COLEC11*
HTLV-I infection (hsa05166)	6	259	3,90E-04	4,91E-03	*CDKN1A, EGR2, ICAM1, IL6, ATF3, IL1R2*
Transcriptional misregulation in cancer (hsa05202)	5	180	5,44E-04	5,63E-03	*CDKN1A, IL6, CXCL8, IL1R2, NR4A3*
Herpes simplex infection (hsa05168)	5	186	6,29E-04	6,09E-03	*IL6, NXF3, CCL2, TNFSF14, SOCS3*
Cytokine-cytokine receptor interaction (hsa04060)	5	265	2,89E-03	2,03E-02	*IL6, CXCL8, CCL2, IL1R2, TNFSF14*
Pathways in cancer (hsa05200)	6	397	3,31E-03	2,13E-02	*CDKN1A, CKS2, GNGT1, IL6, CXCL8, BDKRB1*
**The NHGRI GWAS Catalog**					
Inflammatory bowel disease	9	271	8,03E-07	1,26E-04	*BORCS5, CCL2, CXCL8, ICAM1, IL1R2, NFIL3, PTPRC, SLC11A1, SLC22A4*
Crohn’s disease	6	256	3,68E-04	1,15E-02	*SLC22A4, ICAM1, CCL2, TAGAP, PTPRC, CNNM1*

### Literature data supporting associations revealed for the shared genes

Several associations revealed in the set of shared genes were partially supported by literature data. Three different kinds of evidence were presented: (i) results on animal models, (ii) evidence from the studies of gene polymorphisms and (iii) expression data for any type of prolapse with the same direction of association. Usage of (iii) as an evidence was based on the rationale that heart valve, cartilage, tendon, and bone development share common regulatory pathways^26^. The genes *ADAMTS1* ^12^ *, MYH3* ^27^ and *SERPINE1* ^28^ were up-regulated in POP tissues. Polymorphic variants in the genes *LIN28B* ^29^ and *AGT* ^30^ were associated with POP and mitral valve prolapse (MVP) respectively. *Nfil3-/-* mice developed colitis with high prevalence of rectal prolapse^31^. High expression of *Nlrp3* was found in *Il10-/-*mice with colitis combined with rectal prolapse^32^. The genes *CTSK* ^33^, *MMP19* and *THBS4* ^34^ were over-expressed in MVP. *Egr2-/-* mice had features of human aortic valve disease, in particular excess of proteoglycan deposition and reduction of collagen fibres^35^. This information is given in more detail in Supplementary Table S4.

## Discussion

This study highlighted some important biological processes and putative candidate genes most likely linked with POP development. We present below our vision of POP pathogenesis which is based on our findings and literature data.

The majority of individual gene expression studies in POP were focused on genes related to ECM. Summary data obtained on dozens and even hundreds of patients (Fig. 2) basically supported the common opinion on expression changes of these genes in POP. The results presented in the whole-genome/proteome studies as well as the results of the *in silico* analysis did not confirm these observations. The discrepancies may be linked with the small samples for the whole genome sets, with a highly variable design in terms of investigated tissue, menopausal status, ethnicity, experimental methods and other. Different designs were also used in the studies of candidate genes; however, in larger samples the differences are smoothed out resulting in more accurate parameter estimates, which, in turn, lead to a greater probability to find the desired results. Correction for multiplicity was rarely applied in the studies considering several individual genes; however, in the genome-based approach, any method of selecting the most significant genes was used always (FDR correction, top DEG, top gene sets) with a probability to miss less pronounced but biologically plausible correlations.

Our *in silico* analysis included three independent (AVW, PrM and PM) and two dependent (USL and RL from the same subjects) sets. The design was focused on the search of expression changes in one set and the validation of found differences in other sets. Genes related to ECM-structure were not represented among the sets of shared genes according to the GO results. The shared terms for up-regulated genes revealed the need for response to stimulus, locomotion, adhesion, immune, rhythmic, developmental and some other biological processes which should precede ECM synthesis. In this context, given the substantial histological differences between prolapsed versus non-prolapsed tissues^36, 37^, changes in expression for the most studied ECM genes may be found as a consequence of prolapse rather than an underlying cause^38^. This assumption is in line with the results of a few studies on the role of germline genetic variations in POP. Association studies on the whole-genome level have not revealed genes expected to be linked with connective tissue disorders^29, 39^. Meta-analyses in this field yielded unstable results^40, 41^. Contradictions in the studies of germline genetic variations may partially depend on the underestimation of important risk factors such as perineal trauma in childbirth in the majority of POP genetic association studies^42, 43^. The genetic component of prolapse is rather high^44, 45^ and, given the presence of different kinds of biological activities of proteins encoded by the studied ECM genes (Fig. 2), the question of whether they are only markers or to a certain extent causative genes for POP is still open.

Our findings that in POP tissues genes involved in tissue repair were up-regulated appeared to be unexpected but biologically plausible. Several levels of evidence supported this statement: (i) in all five gene sets under study as well as (ii) in the shared gene set, DEG were enriched with those involved in biological processes implicated in tissue regeneration; (iii) in three PrM sets there was a high co-expression (conceivably co-regulation) of DEG just in POP specimens. Compensatory mechanisms aimed at the recovery of damaged tissues require coordinated regulation of many biological processes with a crucial step being the recruitment of blood cells which mediate inflammatory and immune responses, promoting tissue repair. Inefficiency of these processes may be linked with many reasons, among which the most important are age-related changes. The proportion of women with pelvic floor disorders is dramatically increasing with age reaching up to 10% in women aged 20 to 39 years and up to 50% in women aged 80 years or older^1^.

ECM is constantly being remodeled by degrading and reassembling. In response to injury and other stimuli, remodeling rates increase significantly. ECM degradation products induce inflammation^46^, which is in turn associated with acceleration of proteolytic cascades leading to further destruction of ECM. Inflammation linked or non-linked with infection and other diseases increases with age and age-related clinical conditions. Balanced immune and anti-inflammatory response is crucial for successive regeneration of vaginal tissues. In youth and maturity clinically asymptomatic damage is compensated by repair processes and/or other components of the pelvic floor support mechanism. Aging and menopause are associated with oxidative stress and hormonal disturbances, both conditions strongly exacerbating ECM breakdown processes^47–49^. Tissue remodeling may be successful in youth and maturity but in old age many processes are deregulated and this factor may at least partially explain the delayed onset of the disease with major risk factors (parity and perineal trauma in childbirth) linked to youth.

An interesting finding in our work is that up-regulated genes in prolapsed tissues were enriched with those related to IBD in the GWAS Catalog. IBD includes Crohn’s disease and ulcerative colitis. Persons with constipation-predominant symptoms may suffer from pelvic floor muscular incoordination and failure of normal relaxation of pelvic floor muscles during attempted defecation. GWAS-implicated variants lie on genes that may be linked with immune-mediated disturbances in physiological homeostasis in both diseases. Other hypothesis interpreting shared susceptibility to both disorders might come from the results of animal studies: IBD frequently co-exists with rectal prolapse which is in turn associated with other types of genital prolapse since rectocele leads to overdistension of the perineal body^50^.

The study has some limitations with the main problem being in a small number of datasets and a small number of samples in these datasets. These data appeared to be insufficient for construction of co-expression networks. The results of the enrichment analysis for the overlapping up-regulated genes with GWAS association signals should be discussed as preliminary. These results raise a question rather than provide an answer on a possible shared genetic component for IBD and POP.

Finally, our analysis provided some in-depth data important for understanding POP pathogenesis. In terms of genetic overlap between IBD and POP, the work has translational impact. The study findings are biologically plausible; however, they require verification in independent studies.

## Materials and Methods

### Selection of studies assessing individual gene expression profile

The final search was performed on 11 January 2017 of the PubMed, EMBASE and Web of Science databases in compliance with PRISMA (Preferred Reporting Items for Systematic Reviews and Meta-Analysis) guidelines^51^. The following keyword terms were used as criteriae for searching: pelvic organ prolapse, vaginal prolapse, genital prolapse, uterovaginal prolapse, uterine prolapse, prolapse of vaginal vault, pelvic floor dysfunction, pelvic floor disorder, cystocele, rectocele in combination with the terms: expression, production, secretion, gene and protein. Additional articles were identified by checking reference lists of relevant articles. We used the following inclusion criteria. The article had to be published in English and had to have evaluated POP-related expression changes in pelvic floor supportive tissues in a case-control study *in vivo*. For overlapping studies, we selected those with larger number of subjects.

### Microarray data processing

Results of two studies submitted to the repository Gene Expression Omnibus (GEO) were processed with GEO2R – an interactive web tool which exploits Limma R packages from Bioconductor project^17^ for comparison of user-defined groups of samples under the same experimental conditions (http://www.ncbi.nlm.nih.gov/geo/geo2r/). The use of the ‘Value distribution’ option showed that the data have been normalized and therefore cross-comparable.

### Gene set enrichment analysis

We generated gene sets of DEG from the GEO2R data by setting the cut-off *P* – value < 0.05 (without correction for multiplicity) and fold change ≥2.0. These gene sets were treated with KOBAS 3.0 resource^7^ for Gene Ontology (GO) enrichment analysis. The following settings were applied: the minimum number of genes per category was five, while Benjamini and Hochberg false discovery rate (FDR) corrected *P* - value threshold was 0.05. Web server REVIGO was utilized for summarizing GO terms, which was guided by the *P*-value^9^. We took into account the hierarchical structure among GO terms for data interpretation: when a gene is associated with a term, it is automatically associated with its parent terms^8^.

KOBAS 3.0 tool was additionally used for other types of enrichment analyses for shared genes between gene sets under study, namely metabolic pathways analysis (KEGG PATHWAY) and comparison with the NHGRI GWAS Catalog associations.

### Statistical considerations

Our study was performed to identify common molecular features for POP phenotype. Many promising markers selected in a single data set appeared to be not so promising or even non-significant in independent sets (a winner’s curse problem). External/independent validation is recommended for large-scale (OMICS) studies^52^. Taking into account these recommendations, we searched for shared DEG with the same direction (down-regulation or up-regulation) of the association in independent sets.

Data analyses and visualization were conducted with the R statistical software^53^.

### Compliance with ethical standards

As a secondary analysis of public data the study does not require IRB approval.

## Electronic supplementary material

Supplementary Data

Dataset 1

Dataset 2

Dataset 3
