# Supplementary material for: Expression changes in pelvic organ prolapse: a systematic review and in silico study
Source: Sci Rep. 2017 Aug 9;7:7668. doi: 10.1038/s41598-017-08185-6 (PMC5550478; doi:10.1038/s41598-017-08185-6)
Supplement: Supplementary file 1 — Supplementary Data [file 41598_2017_8185_MOESM1_ESM.pdf]

**Expression changes in pelvic organ prolapse: a systematic review and *in silico* study**

Maryam B. Khadzhieva, PhD[a,b], Dmitry S. Kolobkov[a], Svetlana V. Kamoeva, PhD, MD[c], Lyubov E. Salnikova, PhD, Dr.Sci.(Biol.)[a,b*]

[a]Vavilov Institute of General Genetics, Russian Academy of Sciences, 3 Gubkina str., 119333, Moscow, Russia

[b]Dmitry Rogachev National Research Center of Pediatric Hematology, Oncology and Immunology, 1 Samory Mashela str., 117997, Moscow, Russia

[c]Pirogov Russian National Research Medical University, 1 Ostrovitianov str., 117997, Moscow, Russia

Correspondence to: Lyubov E. Salnikova, Institute of General Genetics, Russian Academy of Sciences, 3 Gubkin Street, Moscow 117971, Russia, phone 74991328958, fax 4991328962

E-mail: salnikovalyubov@gmail.com

**Supplementary Table S4** Literature data for shared up-regulated genes associated with prolapse-related conditions

| Gene symbol | Gene name | Number of sets[a] | Literature evidence | Proposed mechanism[b] | Reference |
|---|---|---|---|---|---|
| *ADAMTS1* | ADAM Metallopeptidase With Thrombospondin Type 1 Motif 1 | 2 | In microarray analysis, the *ADAMTS1* gene was up-regulated 4-fold in the vaginal wall from women with POP (n=5) in comparison with controls (n=5). | This gene is likely to be necessary for normal growth and organ morphology and function (http://www.genecards.org/). | Stratford et al., 2005 |
| *AGT* | Angiotensinogen | 2 | *AGT* M235T polymorphism was associated with mitral valve prolapse. | Angiotensin II can induce proliferation and differentiation of fibroblasts through the involvement of extracellular signal-regulated kinases proteins. Angiotensin-2 acts directly on vascular smooth muscle as a potent vasoconstrictor. | Chou et al., 2002; Fatini et al., 2008; Lung et al., 2008 |
| *CTSK* | Cathepsin K | 2 | Increased expression of CTSK has been reported in human myxomatous mitral valve disease (myxomatous degeneration is the pathological substrate of mitral valve prolapse). | CTSK is involved in osteoclastic bone resorption and may participate in the disorder of bone remodeling. It may play an important role in extracellular matrix degradation (http://www.genecards.org/). | Rabkin et al., 2001 |
| *EGR2* | Early Growth Response 2 | 2 | Defective aortic valves (AoVs) in *Egr2 -/-* mice had features of human AoV disease, in particular excess of proteoglycan deposition and reduction of collagen fibres. | *EGR2* gene controls early myelination of the peripheral nervous system (http://www.genecards.org/). *EGR2* activation in interstitial cells of the AoV leads to *Col1a1* and *Col3a1* up-regulation. | Odelin et al., 2014 |
| *LIN28B* | Protein lin-28 homologue B | 2 | SNP linked to the *LIN28B* gene was associated with POP and stress urinary incontinence in the PheWAS Catalog. | The *LIN28* (*LIN28A* and *LIN28B*) genes may influence myelination in peripheral nervous system thus contributing to insufficient innervation with subsequent POP development. | Salnikova et al., 2016 |

| | | | | | |
|---|---|---|---|---|---|
| *MMP19* | Matrix Metallopeptidase 19 | 2 | *MMP19* was over-expressed in specimens from patients with mitral valve prolapse (n=7) compared with control specimens (from non-beating heart-tissue donors, n=3). | MMP19 is involved in the breakdown of extracellular matrix in normal physiological processes including tissue remodeling. MMP19 is secreted as an inactive proprotein, which is activated upon cleavage by extracellular proteases (http://www.genecards.org/). | Greenhouse et al., 2016 |
| *MYH3* | Myosin Heavy Chain 3 | 2 | *MYH3* was 3.2 times over-expressed in the rectus abdominus muscle of patients with POP (n=15) relative to controls (n=13). | *MYH3* is nvolved in muscle contraction (http://www.genecards.org/). | Hundley et al., 2008 |
| *NFIL3* | Nuclear Factor, Interleukin 3 Regulated | 4 | Approximately 20 and 50% of *Nfil3-/-* mice develop rectal prolapse 20 and 36 wk. of age, respectively. | *Nfil3-/-* mice develop microbiota-dependent spontaneous colitis linked to defects in innate immune response. | Kobayashi et al., 2014 |
| *NLRP3* | NLR Family Pyrin Domain Containing 3 | 2 | NLRP3 inflammasome was upregulated in colonic mucosa of *IL-10-/-* mice and Crohn's patients.  By 6 mo of age all *IL-10-/-* mice had intestinal inflammation and rectal prolapse. | *NLRP3* plays a crucial role in innate immunity and inflammation. | Liu et al., 2016; McCafferty et al., 2000 |
| *SERPINE1* | Serpin Family E Member 1 | | *SERPINE1* significantly increased in stroma from menopausal women with POP (n=8) compared with controls (n=5). | SERPINE1 inhibits active metalloproteinases (MMPs) and suppresses elastic fiber degradation. | Budatha et al., 2013 |
| *THBS4* | Thrombospondin 4 | 2 | *THBS4* was over-expressed in specimens from patients with mitral valve prolapse (n=7) compared with control specimens (from non-beating heart-tissue donors, n=3). | THBS4 is an adhesive glycoprotein that mediates cell-to-cell and cell-to-matrix interactions.  It contributes to local signaling in the developing and adult nervous system (http://www.genecards.org/). | Greenhouse et al., 2016 |

[a]Number of datasets under study, in which the genes were up-regulated. [b]The mechanisms were proposed in cited articles, otherwise specified.

Supplementary Figure S1. Flow diagram of the literature search for the studies of POP-related expression changes

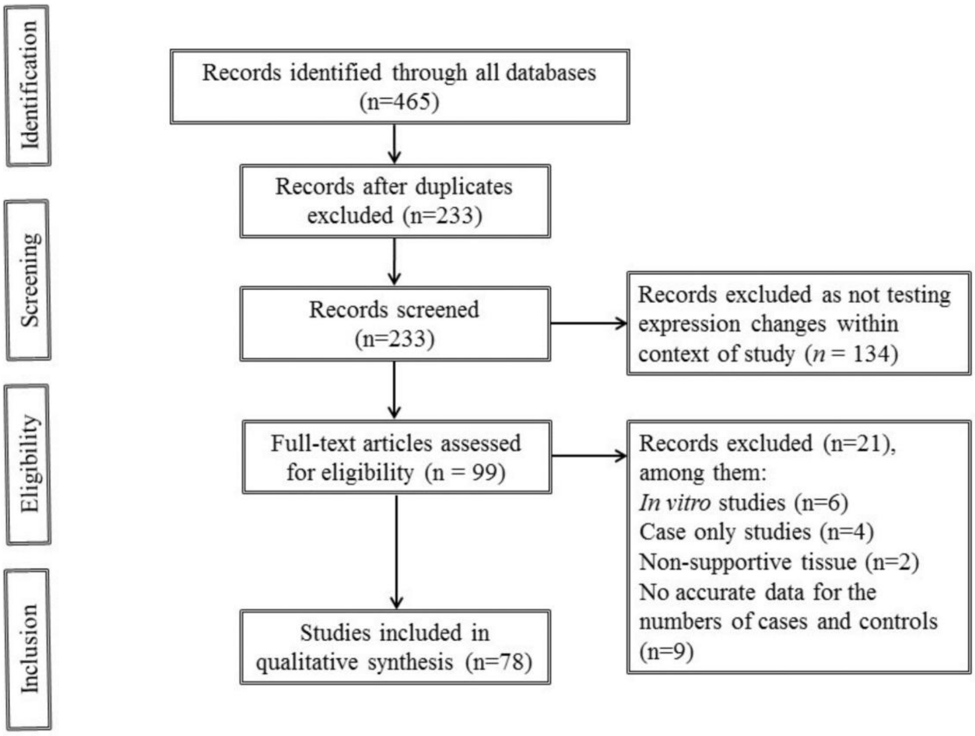

**Supplementary References**


1. Ahn, K. H., Kim, T., Hur, J. Y., Kim, S. H., Lee, K. W. & Kim, Y. T. Relationship between the expression of fibulin-3 and anterior vaginal wall prolapse. *J Obstet Gynaecol.* **32**, 362–366 (2012).
2. Alarab, M., Kufaishi, H., Lye, S., Drutz, H. & Shynlova, O. Expression of extracellular matrix-remodeling proteins is altered in vaginal tissue of premenopausal women with severe pelvic organ prolapse. *Reprod Sci*. **21**, 704–715 (2014).
3. Alarab, M., Bortolini, M. A., Drutz, H., Lye, S. & Shynlova, O. LOX family enzymes expression in vaginal tissue of premenopausal women with severepelvic organ prolapse. *Int Urogynecol J*. **21**, 1397–1404 (2010).
4. Aznal, S. S., Meng, F. G., Nalliah, S., Tay, A., Chinniah, K. & Jamli, M. F. Biochemical evaluation of the supporting structure of pelvic organs in selected numbers of premenopausal and postmenopausal Malaysian women. *Indian J Pathol Microbiol*. **55**, 450–455 (2012).
5. Bai, S. W., Chung, D. J., Yoon, J. M., Shin, J. S., Kim, S. K. & Park, K. H. Roles of estrogen receptor, progesterone receptor, p53 and p21 in pathogenesis of pelvic organ prolapse. *Int Urogynecol J Pelvic Floor Dysfunct.* **16**, 492–496 (2005).
6. Borazjani, A., Kow, N., Harris, S., Ridgeway, B. & Damaser, M. S. Transcriptional regulation of connective tissue metabolism genes in women with pelvic organ prolapse. *Female Pelvic Med Reconstr Surg*. **23**, 44–52 (2017).
7. Boreham, M. K., Wai, C. Y., Miller, R. T., Schaffer, J. I. & Word, R. A. Morphometric properties of posterior vaginal wall in women with pelvic organ prolapse. Am J Obstet Gynecol. 187, 1501–1509 (2002).
8. Bortolini, M. A. et al. Expression of Bone Morphogenetic Protein-1 in vaginal tissue of women with severe pelvic organ prolapse. *Am J Obstet Gynecol*. **204**, 544.e1–8 (2011).
9. Bortolini, M. A. et al. Expression of genes encoding smooth muscle contractile proteins in vaginal tissue of women with and without pelvic organ prolapse. *Neurourol Urodyn*. **31**,109–114 (2012).
10. Brizzolara, S. S., Killeen, J. & Urschitz, J. Gene expression profile in pelvic organ prolapse. *Mol Hum Reprod*. **15**, 59–67 (2009).
11. Budatha, M. et al. Dysregulation of protease and protease inhibitors in a mouse model of human pelvic organ prolapse. *PLoS One*. **8**, e56376 (2013).
12. Budatha, M. et al. Extracellular matrix proteases contribute to progression of pelvic organ prolapse in mice and humans. *J Clin Invest*. **121**, 2048–2059 (2011).
13. Chen, B. H., Wen, Y., Li, H. & Polan, M. L. Collagen metabolism and turnover in women with stress urinary incontinence and pelvic prolapse. *Int Urogynecol J Pelvic Floor Dysfunct.* **13**, 80–87 (2002).
14. Chen, G. D., Chen, Y. C. & Lin, L. Y. Different expression of calpains in the anterior vaginal wall of women with and without uterovaginal prolapse. *J Soc Gynecol Investig.* **11**, 113–117 (2004).
15. Chen, H. Y., Lu, Y., Qi, Y., Bai, W. P. & Liao, Q. P. Relationship between the expressions of mitofusin-2 and procollagen in uterosacral ligament fibroblasts of postmenopausal patients with pelvic organ prolapse. *Eur J Obstet Gynecol Reprod Biol*. **174,** 141–145 (2014).
16. Chen, Y., Huang, J., Hu, C. & Hua, K. Relationship of advanced glycation end products and their receptor to pelvic organ prolapse. *Int J Clin Exp Pathol*. **8**, 2288–2299 (2015).

17. Choy, K. W. et al. High isoprostane level in cardinal ligament-derived fibroblasts and urine sample of women with uterine prolapse. *BJOG*. **115**, 1179–1183 (2008).
18. Connell KA, Guess MK, Tate A, Andikyan V, Bercik R, Taylor HS. Diminished vaginal HOXA13 expression in women with pelvic organ prolapse. *Menopause*. **16**, 529–533 (2009a).
19. Connell, K. A., Guess, M. K., Chen, H., Andikyan, V., Bercik, R. & Taylor, H. S. HOXA11 is critical for development and maintenance of uterosacral ligaments and deficient inpelvic prolapse. *J Clin Invest.* **118**, 1050–1055 (2008).
20. Connell, K.A., Guess, M. K., Chen, H. W., Lynch, T., Bercik, R. & Taylor, H. S. HOXA11 promotes fibroblast proliferation and regulates p53 in uterosacral ligaments. *Reprod Sci*. **16**, 694–700 (2009b).
21. Copas, P., Bukovsky, A., Asbury, B., Elder, R. F. & Caudle, M. R. Estrogen, progesterone, and androgen receptor expression in levator ani muscle and fascia. *J Womens Health Gend Based Med*. **10**, 785–795 (2001).
22. Dietrich, W. et al. Relaxin and gonadal steroid receptors in uterosacral ligaments of women with and withoutpelvic organ prolapse. *Int Urogynecol J.* **23**, 495–500 (2012).
23. Dviri, M., Leron, E., Dreiher, J., Mazor, M. & Shaco-Levy, R. Increased matrix metalloproteinases-1,-9 in the uterosacral ligaments and vaginal tissue from women with pelvic organ prolapse. *Eur J Obstet Gynecol Reprod Biol*. **156**, 113–117 (2011).
24. Eser, A. et al. Is there a relationship between pelvic organ prolapse and tissue fibrillin-1 levels? *Int Neurourol J*. **9**, 164–170 (2015).
25. Ewies, A. A., Al-Azzawi, F. & Thompson, J. Changes in extracellular matrix proteins in the cardinal ligaments of post-menopausal women with or without prolapse: a computerized immunohistomorphometric analysis. *Hum Reprod*. **18**, 2189–2195 (2003).
26. Ewies, A. A., Thompson, J. & Al-Azzawi, F. Changes in gonadal steroid receptors in the cardinal ligaments of prolapsed uteri: immunohistomorphometric data. *Hum Reprod*. **19**, 1622–1628 (2004).
27. Fatini, C. et al. AGT and ACE genes influence classic mitral valve prolapse predisposition in Marfan patients. *Int J Cardiol*. **123**, 293–297 (2008).
28. Gabriel, B. et al. Increased expression of matrix metalloproteinase 2 in uterosacral ligaments is associated with pelvic organ prolapse. *Int Urogynecol J Pelvic Floor Dysfunct*. **17**, 478–482 (2006).
29. Gabriel, B. et al. Uterosacral ligament in postmenopausal women with or without pelvic organ prolapse. *Int Urogynecol J Pelvic Floor Dysfunct.* **16**, 475–479 (2005).
30. Goepel, C. Differential elastin and tenascin immunolabeling in the uterosacral ligaments in postmenopausal women with and without pelvic organ prolapse. *Acta Histochem.* **110**, 204–209 (2008).
31. Greenhouse, D. G., Murphy, A., Mignatti, P., Zavadil, J., Galloway, A. C. & Balsam, L. B. Mitral valve prolapse is associated with altered extracellular matrix gene expression patterns. *Gene*. **586**, 56–61 (2016).
32. Han, L., Wang, L., Wang, Q., Li, H. & Zang, H. Association between pelvic organ prolapse and stress urinary incontinence with collagen. *Exp Ther Med*. **7**, 1337–1341 (2014).
33. He, K., Niu, G., Gao, J., Liu, J. X. & Qu, H. MicroRNA-92 expression may be associated with reduced estrogen receptor β1 mRNA levels in cervical portion of uterosacral ligaments in women with pelvic organ prolapse. *Eur J Obstet Gynecol Reprod Biol.* **198**, 94–99 (2016).

34. Hundley, A. F., Yuan, L. & Visco, A. G. Gene expression in the rectus abdominus muscle of patients with and without pelvic organ prolapse. *Am J Obstet Gynecol*. **198**, 220.e1–7 (2008).
35. Hundley, A. F., Yuan, L., Visco, A. G. Skeletal muscle heavy-chain polypeptide 3 and myosin binding protein H in the pubococcygeus muscle in patients with and without pelvic organ prolapse. *Am J Obstet Gynecol*. **194**, 1404–1410 (2006).
36. Iwahashi, M. & Muragaki, Y. Decreased type III collagen expression in human uterine cervix of prolapse uteri. *Exp Ther Med.* **2**, 271–274 (2011).
37. Jackson, S. R., Avery, N. C., Tarlton, J. F., Eckford, S. D., Abrams, P. & Bailey, A. J. Changes in metabolism of collagen in genitourinary prolapse. *Lancet*. **347**, 1658–1661 (1996).
38. Jeon, M. J. et al. MicroRNA-30d and microRNA-181a regulate HOXA11 expression in the uterosacral ligaments and are overexpressed in pelvic organ prolapse. *J Cell Mol Med.* **19**, 501–509 (2015).
39. Jung, H. J., Jeon, M. J., Yim, G. W., Kim, S. K., Choi, J. R. & Bai, S. W. Changes in expression of fibulin-5 and lysyl oxidase-like 1 associated with pelvic organ prolapse. *Eur J Obstet Gynecol Reprod Biol.* **145**, 117–122 (2009).
40. Karam, J. A., Vazquez, D. V., Lin, V. K. & Zimmern, P. E. Elastin expression and elastic fibre width in the anterior vaginal wall of postmenopausal women with and without prolapse. *BJU Int*. **100**, 346–350 (2007).
41. Klutke, J. et al. Decreased endopelvic fascia elastin content in uterine prolapse. *Acta Obstet Gynecol Scand*. **87**, 111–115 (2008).
42. Kobak, W., Lu, J., Hardart, A., Zhang, C., Stanczyk, F. Z. & Felix, J. C. Expression of lysyl oxidase and transforming growth factor beta2 in women with severe pelvic organ prolapse. *J Reprod Med*. **50**, 827–831 (2005).
43. Kobayashi, T. et al. NFIL3-deficient mice develop microbiota-dependent, IL-12/23-driven spontaneous colitis. *J Immunol*. **192**, 1918–1927 (2014).
44. Kow, N., Ridgeway, B., Kuang, M., Butler, R. S & Damaser, M. S. Vaginal expression of LOXL1 in premenopausal and postmenopausal women withpelvic organ prolapse. *Female Pelvic Med Reconstr Surg.* **22**, 229–235 (2016).
45. Lang, J. H., Zhu, L., Sun, Z. J. & Chen, J. Estrogen levels and estrogen receptors in patients with stress urinary incontinence and pelvic organ prolapse. *Int J Gynaecol Obstet.* **80**, 35–39 (2003).
46. Leegant, A. et al. Transforming growth factor β1 and extracellular matrix protease expression in the uterosacral ligaments of patients with and without pelvic organ prolapse. *Female Pelvic Med Reconstr Surg.* **21**, 53–58 (2015).
47. Li, B. S., Hong, L., Min, J., Wu, D. B., Hu, M. & Guo, W. J. The expression of glutathione peroxidase-1 and the anabolism of collagen regulation pathway transforming growth factor-beta1-connective tissue growth factor in women with uterine prolapse and the clinic significance. *Clin Exp Obstet Gynecol.* **40**, 586–590 (2013).
48. Liang, C. C., Huang, H. Y. & Chang, S. D. Gene expression and immunoreactivity of elastolytic enzymes in the uterosacral ligaments from women with uterine prolapse. *Reprod Sci.* **19**, 354–359 (2012).
49. Liang, C. C., Huang, H. Y., Tseng, L. H., Chang, S. D., Lo, T. S. & Lee, C. L. Expression of matrix metalloproteinase-2 and tissue inhibitors of metalloproteinase-1 (TIMP-1, TIMP-2 and TIMP-3) in women with uterine prolapse but without urinary incontinence. *Eur J Obstet Gynecol Reprod Biol*. **153**, 94–98 (2010).

50. Liapis, A., Bakas, P., Pafiti, A., Frangos-Plemenos, M., Arnoyannaki, N. & Creatsas, G. Changes of collagen type III in female patients with genuine stress incontinence and pelvic floor prolapse. *Eur J Obstet Gynecol Reprod Biol*. **97**, 76–79 (2001).
51. Lin SY, Tee YT, Ng SC, Chang H, Lin P, Chen GD. Changes in the extracellular matrix in the anterior vagina of women with or without prolapse. *Int Urogynecol J Pelvic Floor Dysfunct*. 18, 43–48 (2007).
52. Liu, L. et al. The pathogenic role of NLRP3 inflammasome activation in inflammatory bowel diseases of both mice and humans. *J Crohns Colitis*. doi: 10.1093/ecco-jcc/jjw219 (2016).
53. Meijerink, A. M., van Rijssel, R. H. & van der Linden, P. J. Tissue composition of the vaginal wall in women with pelvic organ prolapse. *Gynecol Obstet Invest.* **75**, 21–27 (2013).
54. Moalli, P. A., Shand, S. H., Zyczynski, H. M., Gordy, S. C. & Meyn, L. A. Remodeling of vaginal connective tissue in patients with prolapse. *Obstet Gynecol*. **106**, 953–963 (2005).
55. Moon, Y. J., Bai, S. W., Jung, C. Y. & Kim, C. H. Estrogen-related genome-based expression profiling study of uterosacral ligaments in women with pelvic organ prolapse. *Int Urogynecol J*. **24**, 1961–7 (2013).
56. Moon, Y. J., Choi, J. R., Jeon, M. J., Kim, S. K. & Bai, S. W. Alteration of elastin metabolism in women with pelvic organ prolapse. *J Urol*. **185**, 1786–1792 (2011).
57. Mosier, E., Lin, V. K. & Zimmern, P. Extracellular matrix expression of human prolapsed vaginal wall. *Neurourol Urodyn*. **29**, 582–586 (2010).
58. Odelin, G. et al. Loss of Krox20 results in aortic valve regurgitation and impaired transcriptional activation of fibrillar collagen genes. *Cardiovasc Res*.104, 443–455 (2014).
59. Phillips, C. H., Anthony, F., Benyon, C., Monga, A. K. Collagen metabolism in the uterosacral ligaments and vaginal skin of women with uterine prolapse. *BJOG*. **113**, 39–46 (2006).
60. Rabkin, E., Aikawa, M., Stone, J. R., Fukumoto, Y., Libby, P. & Schoen, F. J. Activated interstitial myofibroblasts express catabolic enzymes and mediate matrix remodeling in myxomatous heart valves. *Circulation*. **104,** 2525–2532 (2001).
61. Rhee, S. H. al. Pelvic organ prolapse is associated with alteration of sphingosine-1-phosphate/Rho-kinase signalling pathway in human vaginal wall. *J Obstet Gynaecol.* **35**, 726–732 (2015).
62. Saatli, B., Kizildag, S., Cagliyan, E., Dogan, E. & Saygili, U. Alteration of apoptosis-related genes in postmenopausal women with uterine prolapse. *Int Urogynecol J*. **25**, 971–977 (2014).
63. Salnikova, L. E., Khadzhieva, M. B. & Kolobkov, D. S. Biological findings from the PheWAS catalog: focus on connective tissue-related disorders (pelvic floor dysfunction, abdominal hernia, varicose veins and hemorrhoids). *Hum Genet*. **135**, 779–795 (2016).
64. Shi, Z., Zhang, T., Zhang, L., Zhao, J., Gong, J. & Zhao, C. Increased microRNA-221/222 and decreased estrogen receptor α in the cervical portion of the uterosacral ligaments from women with pelvic organ prolapse. *Int Urogynecol J.* **23**, 929–934 (2012).
65. Shynlova, O., Bortolini, M. A. & Alarab, M. Genes responsible for vaginal extracellular matrix metabolism are modulated by women's reproductive cycle and menopause. *Int Braz J Urol.* **39**, 257–267 (2013).

66. Söderberg, M. W., Byström, B., Kalamajski, S., Malmström, A. & Ekman-Ordeberg, G. Gene expressions of small leucine-rich repeat proteoglycans and fibulin-5 are decreased in pelvic organ prolapse. *Mol Hum Reprod*. **15**, 251–257 (2009).
67. Song, Y., Hong, X., Yu, Y. & Lin, Y. Changes of collagen type III and decorin in paraurethral connective tissue from women with stress urinary incontinence and prolapse. *Int Urogynecol J Pelvic Floor Dysfunct*. **18**, 1459–1463 (2007).
68. Stratford, R. R., Baumann, S. S., Jamroz, R. C., Kuehl, T. J., Shull, B. L. & Pierce, L. M. Poster 14: Histology and differential mRNA expression in vaginal connective tissue of women with pelvic relaxation. *Journal of Pelvic Medicine & Surgery*. **11**, S32 (2005).
69. Strinic, T., Vulic, M., Tomic, S., Capkun, V., Stipic, I. & Alujevic, I. Matrix metalloproteinases-1, -2 expression in uterosacral ligaments from women withpelvic organ prolapse. *Maturitas*. **64**, 132–135 (2009).
70. Sun, M. J., Cheng, Y. S., Sun, R., Cheng, W. L. & Liu, C. S. Changes in mitochondrial DNA copy number and extracellular matrix (ECM) proteins in the uterosacral ligaments of premenopausal women with pelvic organ prolapse. *Taiwan J Obstet Gynecol*. **55**, 9–15 (2016).
71. Takacs, P., Gualtieri, M., Nassiri, M., Candiotti, K., Fornoni, A. & Medina, C. A. Caldesmon expression is decreased in women with anterior vaginal wall prolapse: a pilot study. *Int Urogynecol J Pelvic Floor Dysfunct*. **20**, 985–990 (2009).
72. Takacs, P., Gualtieri, M., Nassiri, M., Candiotti K., Fornoni, A. & Medina, C. A. Differential expression of smooth muscle regulatory proteins in the uterosacral ligaments of women with uterine prolapse. *Am J Obstet Gynecol*. **202**, 620.e1–5 (2010a).
73. Takacs, P., Nassiri, M., Candiotti, K., Yang, J., Yavagal, S. & Medina, C. A. Differential expression of fibulins in the uterosacral ligaments of women with uterine prolapse. *Arch Gynecol Obstet.* **282**,389–394 (2010b).
74. Usta, A., Guzin, K., Kanter, M., Ozgül, M. & Usta, C. S. Expression of matrix metalloproteinase-1 in round ligament and uterosacral ligament tissue from women with pelvic organ prolapse. *J Mol Histol*. **45**, 275–281 (2014).
75. Vetuschi, A. et al. Changes in muscularis propria of anterior vaginal wall in women with pelvic organ prolapse. *Eur J Histochem*. **60**, 2604 (2016).
76. Vulic, M., Strinic, T., Tomic, S., Capkun, V., Jakus, I. A. & Ivica, S. Difference in expression of collagen type I and matrix metalloproteinase-1 in uterosacral ligaments of women with and without pelvic organ prolapse. *Eur J Obstet Biol Gynecol Reprod.* **155**, 225–228 (2011).
77. 77. Wang, X., Li, Y., Chen, J., Guo, X., Guan. H. & Li, C. Differential expression profiling of matrix metalloproteinases and tissue inhibitors of metalloproteinases in females with or without pelvic organ prolapse. Mol Med Rep. 10, 2004–2008 (2014).
78. Wen, Y., Ho, J. Y., Polan, M. L. & Chen, B. Expression of apoptotic factors in vaginal tissues from women with urogenital prolapse. *Neurourol Urodyn*. **30**, 1627–1632 (2011).
79. Yılmaz, N. et al. HOXA11 and MMP2 gene expression in uterosacral ligaments of women with pelvic organ prolapse. *J Turk Ger Gynecol Assoc*. **15**, 104–108 (2014).
80. Yucel, N. et al. Immunohistochemical analysis of connective tissue in patients with pelvic organ prolapse. *J Mol Histol.* **44**, 97–102 (2013).
81. Zbucka-Kretowska, M. et al. Expression of estrogen receptors in the pelvic floor of pre- and post-menopausal women presenting pelvic organ prolapse. Folia Histochem Cytobiol. 49, 521–527 (2011).

82. Zhao, B. et al. Interferon-γ and its pathway-associated gene expression in the vaginal tissue of premenopausal females with pelvic organ prolapse. *Exp Ther Med*. **8**, 1145–1149 (2014).
83. Zhao, B. H. & Zhou, J. H. Decreased expression of elastin, fibulin-5 and lysyl oxidase-like 1 in the uterosacral ligaments of postmenopausal women with pelvic organ prolapse. *J Obstet Gynaecol Res*. **38**, 925–931 (2012).
84. Zhou, L. et al. Biomechanical properties and associated collagen composition in vaginal tissue of women with pelvic organ prolapse. *J Urol*. **188**, 875–880 (2012).
85. Zhou, Y., Ling, O. & Bo, L. Expression and significance of lysyl oxidase-like 1 and fibulin-5 in the cardinal ligament tissue of patients with pelvic floor dysfunction. *J Biomed Res*. **27**, 23–28 (2013).
86. Zhu, L., Lang, J., Jiang, F., Jiang, X. & Chen, J. Vasoactive intestinal peptide in vaginal epithelium of patients with pelvic organ prolapse and stress urinary incontinence. *Int J Gynaecol Obstet*. **105**, 223–225 (2009).
87. Zhu, L., Lang, J., Jiang, X., Jiang, F., Chen, J. & Wong, F. Neuropeptide Y expression in vaginal epithelium of women with pelvic organ prolapse and stress urinary incontinence. *Int J Gynaecol Obstet*. **102**, 65–68 (2008).
